# Treatment of the diabetic foot – to amputate or not?

**DOI:** 10.1186/1471-2482-14-83

**Published:** 2014-10-24

**Authors:** Elroy P Weledji, Pius Fokam

**Affiliations:** Department of Surgery, Faculty of Health Sciences, University of Buea, PO Box 126, Limbe, S W Region Cameroon

**Keywords:** Diabetic foot, Infection, Neuropathy, Ischaemia, Treatment, Amputation

## Abstract

**Background:**

Diabetic foot infections are a frequent clinical problem. About 50% of patients with diabetic foot infections who have foot amputations die within five years. Properly managed most can be cured, but many patients needlessly undergo amputations because of improper diagnostic and therapeutic approaches.

**Discussion:**

The article debates the pros and cons of amputation of the diabetic foot. The thesis is that if the guidelines on the management of the diabetic foot are followed primary amputation is only necessary for the unsalvageable diabetic foot. This approach would reduce the incidence of lower limb amputations in diabetic patients.

**Summary:**

We favour the argument that a structured clinical and vascular assessment would help clinical decision- making as to which patients to hospitalize, which to send for imaging, or for whom to recommend surgical interventions. Endovascular procedures are the future in the treatment of diabetic arterial disease and hence the diabetic foot.

## Background

Foot ulcers affect one in ten diabetics during their lifetime [1]. Patients with diabetes have increased risk of lower-extremity amputations and the main cause is diabetic peripheral arterial disease accelerated by the direct damage to the nerves and blood vessels by high blood glucose levels. Wound healing is also impaired from affected collagen synthesis [2, 3]. Diabetic vascular disease has three main components: arteritis and small vessel thrombosis; neuropathy (possibly ischaemic in cause); and large vessel atherosclerosis. In combination these are almost bound to cause problems in the weight- bearing areas. The diabetic foot ulcers are often deeper and more frequently infected than other leg ulcers reflecting the severe end vessel ischaemia and opportunistic infection which is the common experience of the diabetic [1–4]. Factors, such as age and the duration of the disease will increase its incidence and risk of death from uncontrolled infection [4, 5]. Once tissue damage has occurred in the form of ulceration or gangrene, the aim is preservation of viable tissue, but the two main threats are infection and ischaemia [3]. Ulcers should not be automatically treated with antibiotics since although as open chronic wounds there may be many commensal organisms, about half are not infected [3–5]. Several foot-ulcer classification methods have been proposed in order to organize the proposed appropriate treatment plan but none have been universally accepted. The Wagner- Meggitt classification is based mainly on wound depth and consists of 6 wound grades (Table 1) [6]. The University of Texas system grades the ulcers by depth, then stages them by the presence or absence of infection and ischaemia [6, 7]. As there is the need for rapid and more appropriate therapy to facilitate healing, the international working group on the diabetic foot proposed the PEDIS classification which grades the wound on a 5- feature basis: perfusion (arterial supply), extent (area), depth, infection and sensation [1]. They also classified diabetic foot infections into four grades: Grade 1 (no infection; Grade 2 (mild) in subcutaneous tissue only; Grade 3 (moderate) with extensive erythema and infection of deeper tissue and Grade 4 (severe) with systemic inflammatory response indicating severe infection (Table 2) [1–4, 7]. Most diabetic foot infections require some surgical intervention, ranging from minor (debridement) to major interventions including amputation. The main emphasis of the current international guidelines on the management of the diabetic foot is prevention, early recognition and treatment. Prevention of the diabetic foot entails controlling diabetes, smoking, obesity; daily foot checks, removing callosity (neuropathic foot), daily moisturizing, regular toenail cutting, and well fitted footwear [8].

**Table 1:** **The Wagner-Meggitt classification**

Grade 0	Grade 1	Grade 2	Grade 3	Grade 4	Grade 5
***Intact skin***	*superficial ulcer*	*deep ulcer to tendon*, *bone*, *or joint*	*deep ulcer with abscess or osteomyelitis*	*forefoot gangrene*	*whole foot gangrene*

**Table 2:** **Classification of diabetic foot infection** [1]

Grade 1	Grade 2	Grade 3	Grade 4
**No infection**	**Mild**	**Moderate**	**Severe**
*No signs or symptoms of infection*	*Superficial*, *limited in size and depth*	*Deeper or more extensive*	*Systemic signs or metabolic pertubation*

The thesis is that if the guidelines on the management of the diabetic foot are followed primary amputation is only necessary for the unsalvageable diabetic foot (Table 3). Endovascular procedures are the future in the treatment of diabetic arterial disease and hence the diabetic foot.

**Table 3:** **Summary of indications for conservative surgical approach or primary amputation**

Debridement/minor amputation	Primary amputation
*Good blood supply to foot but infected*	*wet gangrene* (*infection* + *ischaemia*)
*Small vessel disease and gangrenous toes*	*life*-*threatening sepsis*
*Successful surgical bypass*	*extensive muscle necrosis*
*Neuropathic foot with little arterial disease*	*revascularisation technically impossible, bed*-*ridden patients*/*functionally useless limb*
*Osteomyelitis with little arterial disease*	

## Discussion

### Arguments for primary amputation

1. Natural history of disease

**Figure 1:**
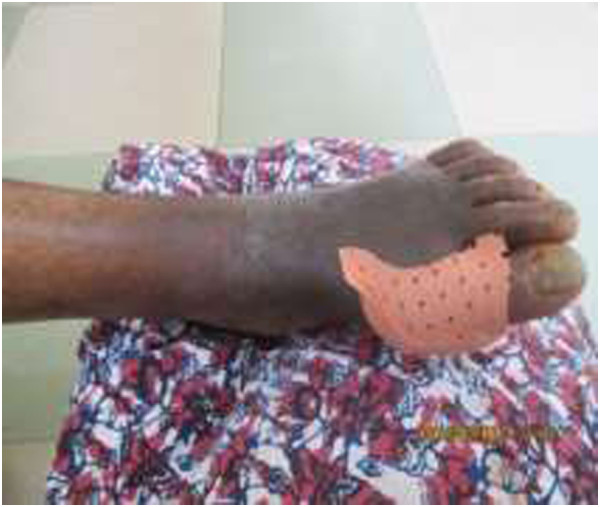
**‘Wet’ gangrene in diabetic patient with peripheral vascular disease (with permission).**

The aim of primary amputation is to relieve pain and achieve rapid and successful mobility with an artificial limb [9]. Peripheral arterial disease is an independent baseline predictor of the non-healing foot ulcer and along with progressing infection continue to be the main reason for lower extremity amputation (Figure 1) [2, 10]. Although the intact foot may withstand markedly reduced skin perfusion, an ulcerated lesion requires a greatly enhanced blood flow to heal; therefore, many ulcers fail to heal where critical ischaemia exists. The progressive development of an abscess in the presence of ischaemia is an ominous sign as it leads to irreparable tissue damage and amputation [4, 5].

2. Assessment and treatment

As pre-operative arteriographic studies and ankle-brachial pressure index (ABPI) are usually unhelpful in the diabetic foot, transcutaneous oxygen measurements have been found useful in some units but the apparatus is expensive and the results are not infallible [10, 11]. The patient’s symptoms, clinical and radiological (duplex ultrasound scanning) findings would dictate the need and level of amputation, including the poorly- controlled diabetic patient with chronic ischaemia who had a failed angioplasty to improve the circulation to the lower limb [10, 12]. Although bowel gas makes duplex ultrasound scanning less useful in the abdomen, the images obtained are often sufficient to plan intervention without the need to resort to invasive imaging [13]. Digital (conservative) amputations are still rarely successful and secondary amputations are common because of disease progression or a preliminary wrong assessment [14]. In practice, most surgeons inspect and palpate the ischaemic limb pre-operatively and observe the intraoperative bleeding from the severed blood vessels at the time of surgery. Major amputations usually below knee is the gold standard, and should be attempted if there is a reasonable chance that it will succeed. Up to 80% of patients become independently mobile because the knee joint is preserved and also a lighter prosthesis is used [9, 12]. The posterior reconstructive transtibial flap (Burgess) method described in 1968 is frequently used but its disadvantage over the equilateral (skew) flap operation described by Kingsley Robinson in 1982 is the risk of ischaemia in the longer posterior flap and the suture line lying over the end of the tibia [9]. There is no difference in the two amputation methods between the rate of primary healing or the need for higher amputation [12]. More distal amputations in patients with distal small vessel disease or those who have had a successful proximal reconstruction, include the Syme’s (forefoot) amputation, a ray amputation of the metatarsal, a transmetatarsal amputation and amputation of the toe [15].

3. Failed revascularization

The greatest immediate danger to these patients after successful revascularization is the ‘reperfusion syndrome’ caused by the release of toxic metabolites and oxygen free radicals into the systemic circulation from the ischaemic limb [16, 17]. This can cause a profound cardiovascular collapse and with renal and sometimes respiratory failure. For this reason revascularisation should not be used in patients with signs of muscle necrosis. Primary amputation is better. A graft should if possible prevent limb loss for at least 2 years if it is to be considered a success. The 2 year patency rate of distal vascular grafts for experienced vascular units should be in the region of 75% [16, 17]. There is evidence that failed bypasses result in a higher level of amputations and the combined mortality rate of a failed reconstruction followed by amputation may be higher than a primary amputation [17].

### Arguments against primary amputation

1. Natural history of disease

The 5-year mortality in patients with diabetes and critical limb ischaemia is 30% and about 50% of patients with diabetic foot infections who have foot amputations die within five years [1, 3]. The mortality rate is similar to some of the most deadly cancers [18]. Poor treatment can lead to lower extremity amputations. About half of these amputations can be prevented by proper care [19–23]. It is vital that the diabetic condition in patients with infection is urgently controlled, otherwise the vicious cycle of infection leading to the instability of the diabetes and ketosis allows the spread of infection [3]. Patients with a severe infection should be hospitalized immediately as these are often imminently limb-threatening and, in some cases life- threatening [3, 18]. When all or part of a foot has dry gangrene, it may be preferable especially for a patient who is a poor surgical candidate to let the necrotic portions auto-amputate. It may also be best to leave adherent eschar in place, especially on the heel, until it softens enough to be more easily removed, provided that there is no underlying focus of infection [12]. Wet gangrene develops if infection supervenes and this spreads rapidly leading to a severely compromised limb, systemic sepsis and death if there is no intervention [21]. However, the required emergency amputation still carries a high mortality of up to 50% because of severe sepsis and the effects of tissue necrosis [24].

2. Assessment and treatment

The diabetic patient presenting with a foot wound should be assessed at three levels- the patient as a whole, the affected limb and foot and the infected wound [1–4]. The affected limb and foot should be assessed for arterial ischaemia, venous insufficiency, presence of protective sensation, and biomechanical problems.There may be an obvious large wound or ulcer associated with erythema and pyrexia. The presence of any exposed bone and ulcer larger than 2 cm [2] increase the likelihood of osteomyelitis [1, 3]. It is suspected in a patient with an adequate blood supply to the affected foot that has a deep ulcer which would not heal after 6 weeks of appropriate wound care and off-loading [25]. Some diabetic patients who develop neuropathies or osteomyelitis but with little arterial disease may often benefit from surgical debridement or excision and/or prolonged antibiotic therapy for at least 4 weeks, based on the culture and sensitivity of biopsied bone tissue or the curettage of deep tissues [3, 4, 26]. Swab specimens, especially of incompletely debrided wounds provide less accurate results [1, 27].

It is important to distinguish between the ischaemic and the neuropathic foot with respect to management although these factors may co-exist [28]. The neuropathic foot is characterized by warm, dry, bounding pulses as a result of peripheral vasodilatation, callosities, painless penetrating ulcers at pressure points and sites of minor injury, painless necrosis of toes, spreading infection along plantar spaces, general loss of pain and thermal sensation, decrease ankle jerk reflex, tone and power [29, 30]. The ischaemic foot is characterized by cold, absent pulses, dependent rubor, trophic changes, absent callosities, painful ulcers around heels and toes, claudication and rest pain [31].

3. Diabetic foot infection

Diabetic foot infections typically begin in a neuropathic ulceration. An infected diabetic foot with good blood supply would respond to debridement [32]. In neuropathic foot, severe infection is treated with intra-venous antibiotics in hospital and, antiseptics and dressings for ulcers. Necrotic tissue is removed and conservative digital amputations or filleting is sufficient.The surgical approach would optimize the likelihood for healing while attempting to preserve the integrity of the walking surface of the foot [30]. Specialised footwear is used to reduce weight bearing [1, 4]. In ischaemic foot infection is treated by debridement (cleaning the wound, removing pus, dead necrotic tissue and infected bone) [1, 31].

While all wounds are colonized with microorganisms, the presence of infection is defined by findings of inflammation or purulence [1, 3]. There are usually complex polymicrobial infections, but aerobic gram positive cocci is a vital part of diabetic foot infection. A broad-spectrum intra- venous antibiotic and metronidazole for anaerobes are recommended. Antibiotics can usually be discontinued once the clinical signs and symptoms of infection have resolved usually 1–2 weeks for mild infection and 2–3 weeks for moderate to severe infection, and not until the wound has healed. This is to avoid resistance [4]. If the wound is not easily debrided varidase dressing is used, and inadine or granuflex dressing would promote granulation [33, 34]. The use of topical antimicrobials for most clinically uninfected wounds is not advocated for lack of evidence substantiating the benefit over conventional wound care therapy [1, 4, 35]. Several recent systematic reviews have suggested that silver-containing dressings and topical silver were neither better nor worse than control dressings in preventing wound infection and prolong healing [36]. New techniques for wound debridement include low frequency ultrasound therapy, hydrosurgery, monofilament polyester fibre pad and plasma-mediated bipolar radiofrequency ablation [37]. Skin grafting when no infection is present may be required [24].

The diabetic foot infection classification system (Table 2), along with a vascular assessment, would help determine which patients should be hospitalized, which may require special imaging procedures or surgical interventions including amputation [1, 3]. Vascular assessment that reveals small vessel disease with associated gangrenous toes may be successfully treated with debridement and minor amputation [10].

4. Revascularisation

As diabetes is chronic and progressive, it makes sense to have a conservative surgical approach that include surgical revascularization [10]. A successful surgical bypass of larger vessel disease may enable more conservative treatment of the diabetic foot. Revascularisation is, however, considered inappropriate in bedridden patients, in a functionally useless limb, in patients with life threatening sepsis, extensive muscle necrosis and where it is technically impossible. Primary amputation is better in these cases [3, 17].

A percutaneous transluminal angioplasty (PTA) and luminal stenting or arterial reconstruction to improve blood flow would aid healing [13]. Because in most cases ischaemia is secondary to larger vessel artherosclerosis rather than to ‘small vessel disease’, vessels above the knee and below the ankle tend to be relatively spared. Thus lower extremity artherosclerosis can be amenable to angioplasty or vascular bypass [16]. The indications for a PTA in diabetic peripheral arterial disease are classically for disabling claudication and critical limb ischaemia, Patients with non-critical ischaemia (ankle/brachial pressure index (ABPI- 0.4-0.9) can in some cases be successfully treated without a vascular procedure [17]. Although the prevalence of ABI <0.9 in individuals with normal glucose tolerance was 7% and increased to 20.9% with diabetes, care should be taken when interpreting ABPI in diabetics [11]. Arterial calcification of the vessel media renders the vessels incompressible and causes false ‘high’ readings. Toe pressure measurements may be of value. Revascularization by percutaneous transluminal angioplasty (PTA) of short segment disease was feasible in more than 96% of diabetics with critical limb ischaemia (ankle systolic pressure of less than 50 mmHg or the toe pressure of less than 30 mmHg) [13]. Many centres have reported successful use of both aggressive endovascular interventions and distal bypass procedures for more severe vascular disease of the foot. The short-term effects are satisfactory with healing of the foot ulcers and thus diminishing the risk of amputation. However, follow-up is required to ascertain the long-term effects [10, 17, 38]. The feasibility with bypass prosthetic grafting (BPG) is lower but consistent [16]. Studies strongly suggest that early recognition and aggressive surgical drainage of pedal sepsis followed by surgical revascularization is critical to achieving maximal limb salvage of 74% at 5 years in the high risk population [17]. The risks of unsuccessful revascularization leading to limb loss must be weighed against the benefits and the patient informed. However, careful debridement of necrotic, infected diabetic foot wound should not be delayed while awaiting revascularization [3, 10].

Aggressive attempts at foot salvage are justified in diabetic patients with advanced forefoot tissue loss/infection. After procuring adequate arterial tissue perfusion, a less conservative transtarsal (mid-foot) amputations salvaged over half of non-healing transmetatarsal amputations with excellent functional results [39].

5. Postoperative sepsis

Smokers, older patients with longer history of uncontrolled diabetes, and those with gangrenous infections and large ulcers have poorer outcome with amputations [4, 5, 10]. Many patients are elderly with impaired continence and poor hygiene and as a number carry *Clostridium perfringens* in their stools post operative mortality from gas gangrene is high [9]. The major problem is stump infection, which is always caused by the same organisms found in the gangrenous tissues. A swab should therefore be taken from infected lesions in the foot so that appropriate antibiotics can be administered [1]. Normally these are given with the premedication prophylactically unless there is marked infection and cellulitis which require urgent treatment [3].

6. Postoperative amputation pain and rehabilitation

Post- operative amputation pain is mostly due to phantom limb pain (54%) and phantom limb sensation (90-98%) [40]. Phantom limb pain usually continues for more than six months whereas phantom limb sensation (except pain) usually disappears or decreases with time. The true mechanism is not known but many theories overlap a peripheral, spinal and central mechanism. The successful treatment of phantom limb pain is thus difficult and treatment is usually combined and multiple based on the person’s level of pain. These include biofeedback to relieve muscle tension, physical therapy, surgery to remove scar tissue entangling a nerve, transcutaneous electrical nerve stimulation (TENS) of the stump, neurostimulation techniques, medications such as analgesics, neuroleptics, anticonvulsants, antidepressants, beta -blockers and sodium channel blockers [41]. The patient must therefore be properly prepared for surgery psychologically with time being spent on assessment by the physiotherapist and reassurance and encouragement being provided by the surgeons, ward nurses or a successful amputee. The patient should be encouraged to spend periods lying prone to help keep the knee straight post-operatively and avoid fixed flexion deformity. The level of amputation may have to be high enough to ensure adequate healing of the stump [42]. Above Knee amputation (AKA) or ‘transfemoral amputation’ is associated with a much poorer outcome because these patients are more often unwell than those needing a below knee or ‘transtibial amputation’ (BKA). Although AKA is more likely to heal, rehabilitation is less successful [9]. Most elderly patients are not psychologically prepared and rehabilitation is an up-hill task.

## Summary

Many diabetic foot problems are avoidable. Good glycaemic control and patient’s education are essential. The main determinant of which patients with a diabetic foot infection need to be hospitalized is the clinical severity of the infection. With minimal surgical trauma and certain curative effect endovascular procedures is the future in the treatment of diabetic peripheral arterial disease and thence the diabetic foot. It is desirable that a vascular surgeon should assess the diabetic foot as the possibility of revascularization must always be considered and the correct sub-group selected for amputation. Guideline-based care for diabetic foot infections and the employment of multidisciplinary teams would help improve outcome and minimize amputations.
